# Loss of TIMP3 by promoter methylation of Sp1 binding site promotes oral cancer metastasis

**DOI:** 10.1038/s41419-019-2016-0

**Published:** 2019-10-17

**Authors:** Chun-Wen Su, Yu-Chao Chang, Ming-Hsien Chien, Yi-Hsien Hsieh, Mu-Kuan Chen, Chiao-Wen Lin, Shun-Fa Yang

**Affiliations:** 1 Institute of Medicine, Chung Shan Medical University, Taichung, Taiwan; 2 Department of Medical Research, Chung Shan Medical University Hospital, Taichung, Taiwan; 3 School of Dentistry, Chung Shan Medical University, Taichung, Taiwan; 4 Department of Dentistry, Chung Shan Medical University Hospital, Taichung, Taiwan; 5 Graduate Institute of Clinical Medicine, College of Medicine, Taipei Medical University, Taipei, Taiwan; 6 Department of Medical Education and Research, Wan Fang Hospital, Taipei Medical University, Taipei, Taiwan; 7 Institute of Biochemistry, Microbiology and Immunology, Chung Shan Medical University, Taichung, Taiwan; 8 Department of Otorhinolaryngology-Head and Neck Surgery, Changhua Christian Hospital, Changhua, Taiwan; 9 Institute of Oral Sciences, Chung Shan Medical University, Taichung, Taiwan

**Keywords:** Oral cancer, Cell invasion

## Abstract

The tissue inhibitor of metalloproteinase-3 (TIMP3) is the only member of the TIMP family that binds to the extracellular matrix and suppresses cancer cell growth, angiogenesis, migration, and invasion. However, whether the abnormal expression and promoter methylation of TIMP3 facilitates oral cancer metastasis remain unclear. In this study, the DNA methylation levels of TIMP3 CpG islands were assessed through pyrosequencing. Artificial modulation of TIMP3 was performed to explore the role of TIMP3 in tumor metastasis in vitro and in vivo. Our results showed that the suppression of TIMP3 transcription by DNA methylation involves the inhibition of the binding of the transcription factor Sp1 to the TIMP3 promoter as well as the upregulation of DNMT1 and DNMT3B. Functional analyses revealed that TIMP3 overexpression reduced migration and invasion abilities in oral cancer cells and inhibited lymph node metastasis in vivo. Moreover, TIMP3 regulated epithelial–mesenchymal transition by increasing the expression of the epithelial markers and reducing the expression of the mesenchymal markers. In conclusion, our findings suggested that the suppression of TIMP3 by DNA methylation contributes to oral cancer metastasis.

## Introduction

Oral squamous cell carcinoma (OSCC) is the most common malignancy of the head and neck and includes cancers of the lip, tongue, gingiva, floor of the mouth, buccal mucosa, palate, and other intraoral locations. Primary risk factors associated with OSCC include alcohol drinking, betel quid chewing, radiation, and viral infections^1,2^. This type of malignancy has a high potential for local invasion and lymph node metastasis^3,4^, and metastasis is the most vital cause of death. Metastasis of cancer comprises multiple events involving cancer cell migration, cancer cell invasion, epithelial–mesenchymal transition (EMT), angiogenesis, and extracellular matrix (ECM) disruption^5,6^. Various proteinases, such as matrix metalloproteinases (MMPs), cathepsins, and plasminogen activators, are involved in ECM degradation during metastasis^7^.

The tissue inhibitor of metalloproteinase-3 (TIMP3) is a member of the TIMP family. TIMP3 is a 24-kDa secreted protein, and unlike other family members, it binds firmly to the ECM. In addition, TIMP3 demonstrates a broad range of metalloproteinase inhibitory activity against the members of MMPs, a disintegrin and metalloproteinases (ADAM), and ADAM with a thrombospondin domain (ADAM-TS) families^8,9^. TIMP3 acts as a tumor suppressor gene, and decrease of TIMP3 expression has been found in esophageal adenocarcinoma, gastric adenocarcinoma, and clear cell renal cell carcinoma^10,11^. TIMP3 possesses numerous anticancer properties; for example, it exerts an antiangiogenesis effect by blocking the binding of VEGF to the VEGF receptor-2^12^. TIMP3 plays a crucial role in promoting apoptosis in prostate cancer cells and inhibits the migration and invasion abilities of tumor cells in osteosarcoma^13,14^. Loss of expression of one gene can be caused by various mechanisms including genetic or epigenetic alternations. DNA promoter hypermethylation is an epigenetic mechanism that may cause transcriptional silencing by interfering with the binding of transcription factors to DNA promoters^2,15^. Tumor suppressor genes, such as RUNX3, RASSF1A, and CD44, are often silenced in cancer because of promoter hypermethylation^16–18^. In oral cancer, many tumor suppressor genes, including APC, survivin, E-cadherin, MGMT, MLH1, p14ARF, p15INK4B, p16INK4A, RARβ, and RASSF, have been examined for DNA methylation. All these genes have been identified to play a role in carcinogenesis and have been implicated in other tumor types^2,19,20^.

Although studies have identified TIMP3 as a tumor suppressor gene in many cancer types, few reports still exist on whether the abnormal expression and promoter methylation of TIMP3 facilitates oral cancer metastasis. Therefore, in the present study, we investigated the relationship between TIMP3 and oral cancer by analyzing TIMP3 expression in oral cancer cell lines and oral tissues obtained from patients with oral cancer. Furthermore, we examined whether TIMP3 is regulated by the hypermethylation of promoters by analyzing its methylation status in oral tissues and oral cancer cell lines through pyrosequencing. Finally, we determined the function of TIMP3 in oral cancer by transfecting a TIMP3 overexpression vector into oral cancer cells and examining the cell growth, along with their migration, invasion, and adhesion abilities after TIMP3 restoration.

## Results

### Loss of TIMP3 and promoter hypermethylation were frequent in oral cancer

**Fig. 1:**
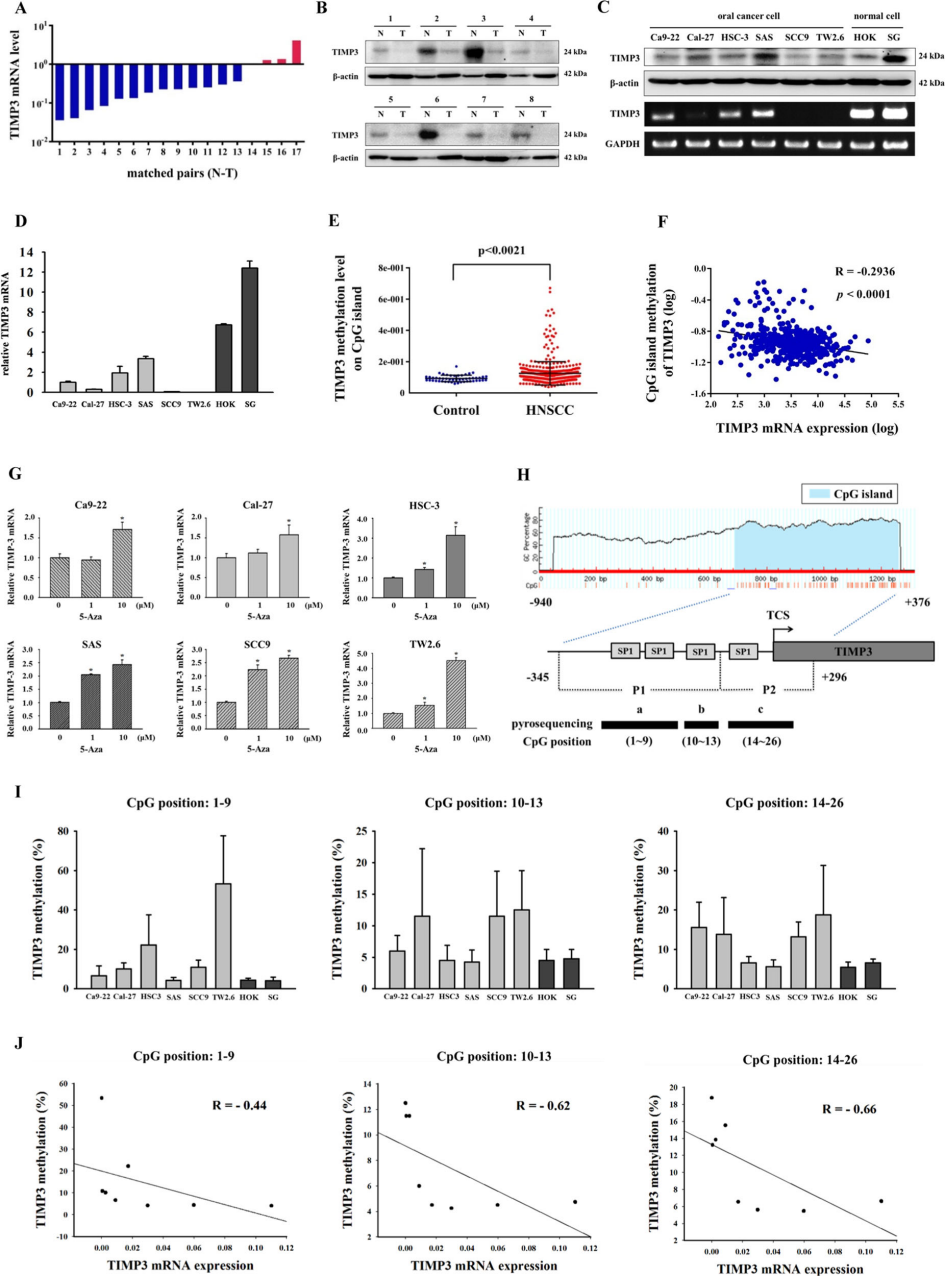
Loss of TIMP3 and promoter hypermethylation was frequent in oral cancer. **a** TIMP3 mRNA levels of oral cancer tissues and corresponding normal tissues. The TIMP3 mRNA levels of corresponding normal tissues were set as 1. Vertical bars, paired samples. N, corresponding normal tissue. T, oral cancer tissue. **b** TIMP3 protein levels of oral cancer tissues and corresponding normal tissues, β-actin was used as loading control. N, corresponding normal tissue. T, oral cancer tissue. **c** TIMP3 protein and mRNA levels of oral cell lines, β-actin and GAPDH was used as loading control. **d** TIMP3 mRNA levels of oral cell lines were detected by real-time PCR, GAPDH was used as internal control. **e** TIMP3 methylation levels of CpG island in HNSCC tissues and normal tissues from MethHC database. **f** The correlation between TIMP3 methylation levels of CpG island and TIMP3 mRNA levels from MethHC database. **g** TIMP3 mRNA levels of oral cancer cell lines after treatment of 5-aza. **h** Overview of the TIMP3 CpG locations. Three sequences a (CpG sites: 1–9), b (CpG sites: 10–13), and c (CpG sites: 14–26) were analyzed by pyrosequencing after amplifying P1 and P2 fragments. **i** The average methylation levels of a, b, and c sequences in oral cell lines. **j** The correlation between average methylation levels of three sequences and TIMP3 mRNA levels in oral cell lines

To determine whether TIMP3 is downregulated in oral cancer, 25 pairs of tissues from patients with oral cancer, including the cancer tissue and its corresponding normal tissue, were used to analyze the TIMP3 expression level. Results showed that TIMP3 mRNA levels decreased in the cancer tissue compared with the normal tissue in 13 patients from 17 pairs of oral tissues (Fig. 1a). The TIMP3 protein level was also downregulated in eight pairs of cancer tissues (Fig. 1b). Furthermore, we evaluated TIMP3 protein and mRNA levels in human OSCC cell lines (Ca9-22, Cal-27, HSC-3, SAS, SCC9, and TW2.6) and normal oral cell lines (HOK and SG). Results showed that TIMP3 was downregulated in the cancer cell lines compared with the normal cell lines (Fig. 1c). Furthermore, the TIMP3 mRNA level in the oral cell lines was analyzed through real-time PCR (Fig. 1d). Loss of gene expression may be caused by the hypermethylation of CpG islands in the promoter regions of tumor suppressor genes^21^. Data on the CpG island methylation level of TIMP3 from the MethHC database revealed that the methylation level was higher in HNSCC tissues than in normal tissues (Fig. 1e). Moreover, a significantly negative correlation existed between the methylation levels of TIMP3 CpG islands and TIMP3 mRNA levels in HNSCC tissues (Fig. 1f). Next, to investigate whether TIMP3 inactivation is caused by DNA hypermethylation, we treated our cancer cell lines with 5-aza-2′-deoxycytidine (5-aza), a DNA methyltransferase inhibitor. After treatment with 5-aza, the TIMP3 mRNA level was elevated in all oral cancer cell lines (Fig. 1g). This result indicated that the loss of TIMP3 in oral cancer cells may be caused by DNA hypermethylation. To further analyze the methylation status of the TIMP3 promoter, the distribution of CpG islands on the TIMP3 promoter (−940 to +376) was predicted using MethPrimer and analyzed through pyrosequencing. The pyrosequencing assay was designed to detect three sequences: a (CpG site 1–9), b (CpG site 10–13), and c (CpG site 14–26). The three sequences were located on the CpG islands of the TIMP3 promoter and covered four Sp1 sites (Fig. 1h). Results showed that the methylation status of the fragments a, b, and c was lower in the normal oral cell lines than in the oral cancer cell lines, whereas the cell lines SCC9 and TW2.6 with lower TIMP3 mRNA expression were highly methylated in fragments b and c (Fig. 1i). Moreover, CpG methylation levels were negatively correlated with mRNA levels in the oral cell lines (Fig. 1j).

### Suppression of TIMP3 by DNA methylation involved inhibition of the binding ability of Sp1 to the TIMP3 promoter and upregulation of DNMTs

**Fig. 2:**
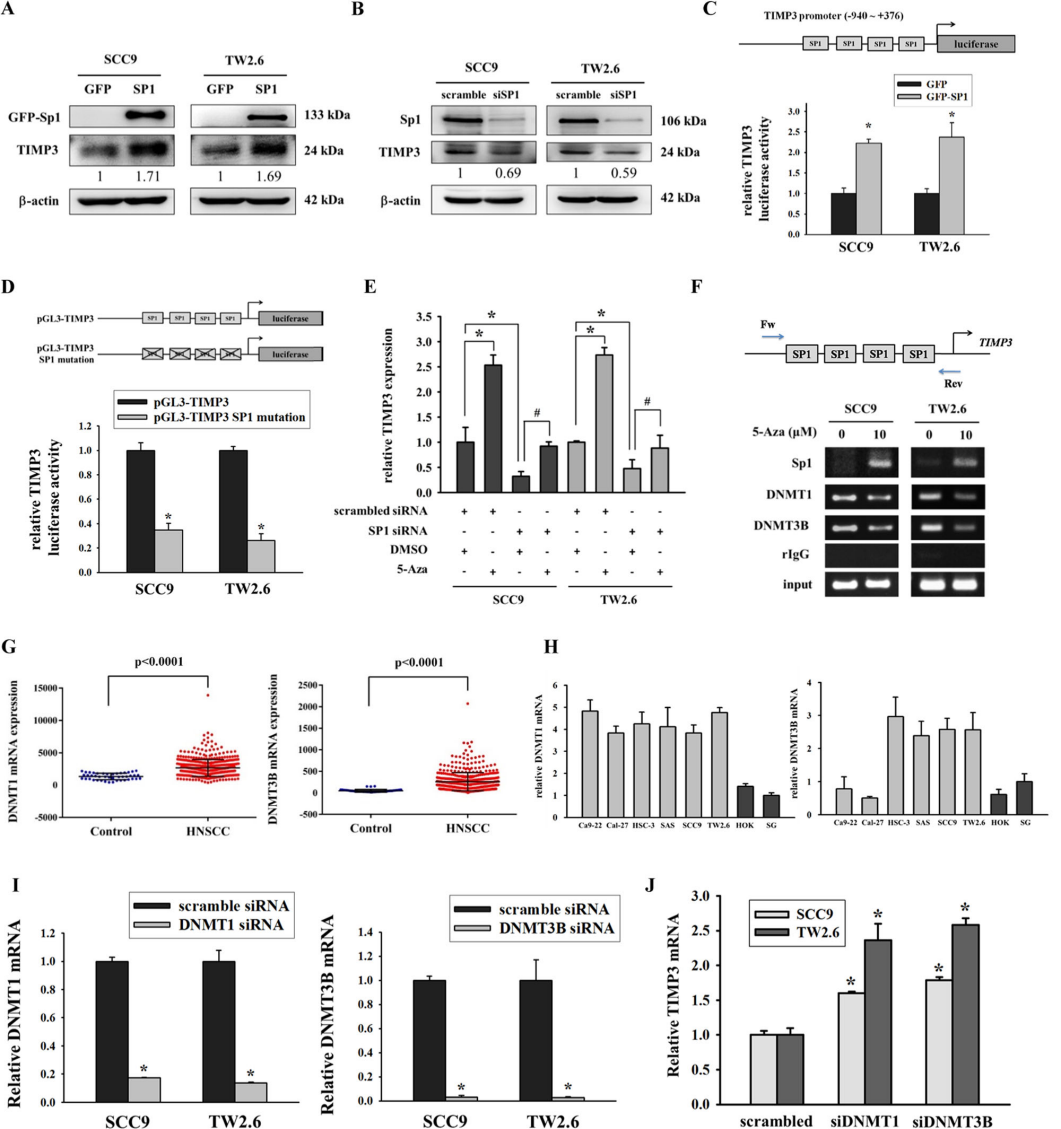
Suppression of TIMP3 by DNA methylation involved inhibition of the binding ability of Sp1 to the TIMP3 promoter and upregulation of DNMTs. **a** Western blot results of Sp1 and TIMP3 after transfection of GFP vector and GFP-SP1 vector, β-actin was used as internal control. **b** Western blot results of Sp1 and TIMP3 after knockdown of Sp1, β-actin was used as internal control. **c** TIMP3 promoter activity after transfection of Sp1 overexpression vector, β-gal was used to normalize transfection efficiency. *p < 0.05 compared with GFP. **d** TIMP3 promoter activity after mutation of the Sp1 binding sites, β-gal was used to normalize transfection efficiency. *p < 0.05 compared with pGL3-TIMP3. **e** SCC9 and TW2.6 cells were transfected with the control siRNA or Sp1 siRNA. After 24 h, cells were treated with the vehicle control (DMSO) or 5-aza (10 μM) for 96 h before total RNA was subjected to qPCR analysis. *p < 0.05 compared with treatment of scrambled siRNA and DMSO. ^#^*p* < 0.05 compared with treatment of Sp1 siRNA and DMSO. **f** SCC9 and TW2.6 cells were treated with the vehicle control (DMSO) or 5-aza (10 μM) for 96 h and were subjected to immunoprecipitation with an antibody against Sp1, DNMT1, and DNMT3B. The precipitates were subjected to PCR amplification using primers directed to Sp1 binding site of the TIMP3 promoter. **g** DNMT1 and DNMT3B levels in HNSCC tissues and normal tissues from TCGA database. **h** DNMT1 and DNMT3B levels in oral cancer cell lines and normal oral cell lines. **i** The mRNA expression of DNMT1 and DNMT3B after transfection of DNMT1 siRNA or DNMT3B siRNA. *p < 0.05 compared with scrambled siRNA. **j** TIMP3 levels after knockdown of DNMT1 or DNMT3B in SCC9 and TW2.6 cells. *p < 0.05 compared with scrambled siRNA

The human TIMP3 promoter contains many binding sites for the transcription factor Sp1^22^. To determine the relationship between Sp1 and TIMP3, an Sp1 overexpression vector or Sp1 siRNA was transfected into SCC9 and TW2.6 cells. Western blot results showed that the protein expression of TIMP3 increased when Sp1 was restored (Fig. 2a) and decreased after Sp1 knockdown (Fig. 2b). To further examine whether the TIMP3 promoter activity is regulated by Sp1, we constructed a luciferase reporter vector that contained the TIMP3 promoter sequence (−940 to +376). The results of the luciferase reporter assay showed that Sp1 expression in SCC9 and TW2.6 cells increased the activity of the human TIMP3 promoter (Fig. 2c). The importance of Sp1 was also confirmed using the site mutation assay. Data revealed that the mutation of all Sp1 sites resulted in decreased TIMP3 promoter activity (Fig. 2d). Loss of genes may be caused through transcriptional silencing by interfering with the binding of transcription factors to the DNA promoter. Real-time PCR data indicated that the siRNA knockdown of Sp1 inhibited the upregulation of TIMP3 mRNA in response to 5-aza (Fig. 2e). SCC9 and TW2.6 cells were also treated with 5-aza, and the binding ability of Sp1 to the TIMP3 promoter was analyzed using the chromatin immunoprecipitation assay. Results indicated that demethylation by 5-aza increased the binding ability of Sp1 and reduced the binding ability of DNA methyltransferases (DNMT1 and DNMT3B) on the TIMP3 promoter (Fig. 2f). The overexpression of DNA methyltransferases (mainly DNMT1 and DNMT3B) in various tumors results in the hypermethylation of tumor suppressor genes^23^. In the present study, we first analyzed the mRNA levels of DNMT1 and DNMT3B from the TCGA database. Results suggested that the mRNA levels of DNMT1 and DNMT3B were increased in HNSCC tissues compared with normal tissues (Fig. 2g). Furthermore, in vitro data showed that DNMTs were increased in oral cancer cell lines, except DNMT3B for Ca9-22 and Cal-27 cell lines (Fig. 2h). In addition to being highly expressed in some oral cancer cell lines, DNMT3A was highly expressed in the normal cell line SG (Fig. S1). To further demonstrate the contribution of DNMTs to the regulation of TIMP3, we knocked down the expression of DNMT1 or DNMT3B by using their own siRNA (Fig. 2i). Data suggested that TIMP3 expression recovered after the knockdown of DNMT1 or DNMT3B (Fig. 2j).

### Restoration of TIMP3 inhibited migration and invasion abilities of oral cancer cells

**Fig. 3:**
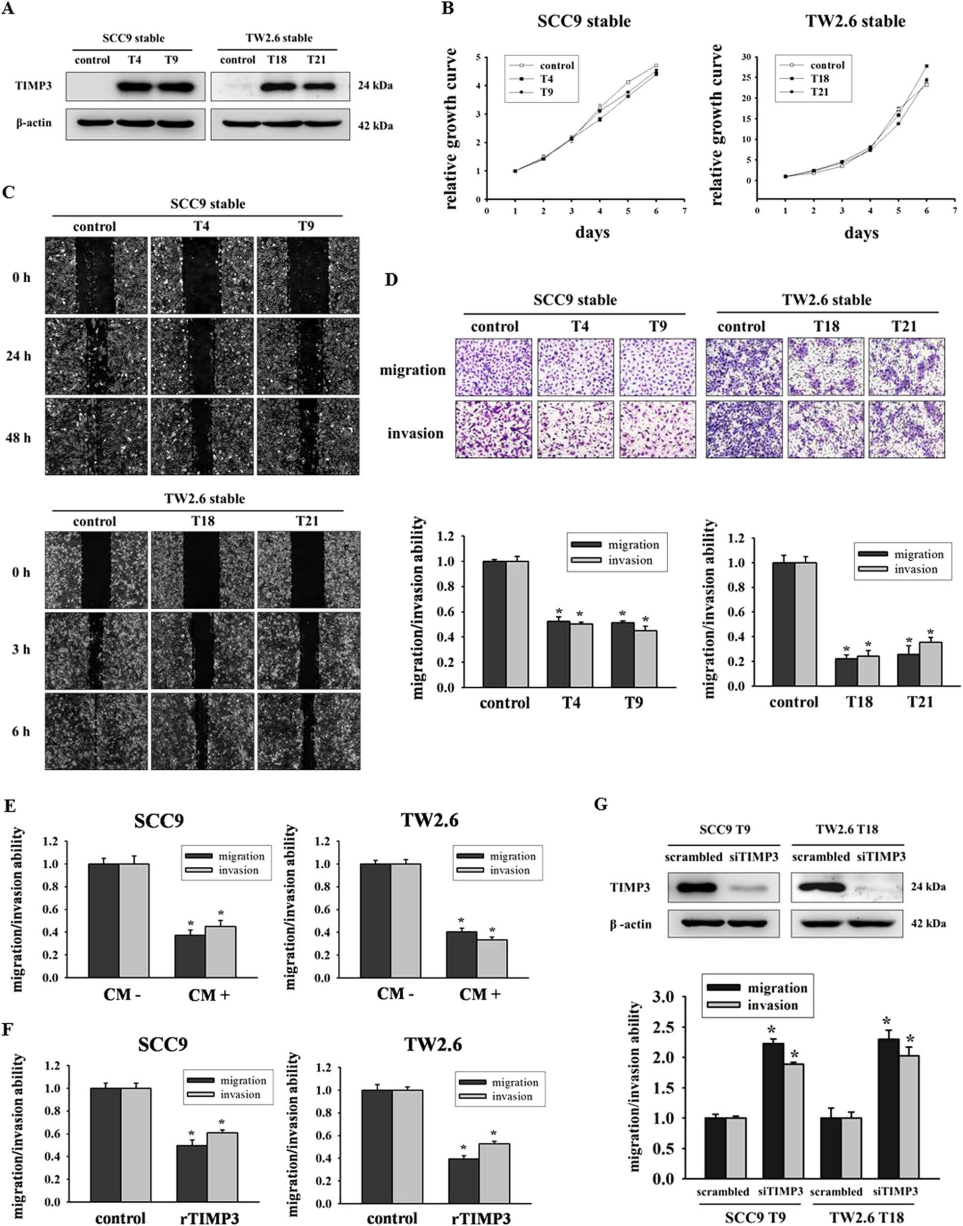
Restoration of TIMP3 inhibited migration and invasion abilities of oral cancer cells. **a** Western blot of SCC9 and TW2.6 stable clones, β-actin was used as internal control. **b** Cell proliferation was analyzed by MTT assay. The cell number in first day was set as 1 and used for normalization. **c** Clones were wounded for 0 h, 24 h, and 48 h (SCC9) or 0 h, 3 h, and 6 h (TW2.6). Phase-contrast pictures of the wounds at three different locations were taken. **d** Migration and invasion abilities were measured after 24 h and 48 h. *p < 0.05 compared with control group. **e** Migration and invasion abilities of SCC9 and TW2.6 cells exposed to their own stable conditioned medium (CM−: control cells; CM+: SCC9-T9 or TW2.6-T18) as chemoattractant, or to **f** recombinant TIMP3 protein (rTIMP3). **g** Migration and invasion abilities of SCC9-T9 and TW2.6-T18 after transfection with scrambled siRNA or TIMP3 siRNA. *p < 0.05 compared with scrambled siRNA

To confirm the expression of the transgene in infected cells, we first performed Western blot analysis on whole cell lysates prepared from stable clones to analyze TIMP3 expression. TIMP3 was overexpressed in SCC9–TIMP3 stable clones (T4 and T9) and TW2.6–TIMP3 stable clones (T18 and T21) compared with control clones (Fig. 3a). Moreover, the results of the MTT assay showed that no significant differences existed between the proliferation of TIMP3 stable clone cells and control clone cells (Fig. 3b). Next, we investigated the motility of TIMP3 stable clones by performing a wound healing assay. The overexpression of TIMP3 reduced cell motility compared with control cells (Fig. 3c). According to the results of the migration and invasion assay, the overexpression of TIMP3 inhibited the migration and invasion abilities of SCC9–TIMP3 and TW2.6–TIMP3 stable clones (Fig. 3d). Subsequently, we investigated both the migration and invasion abilities of SCC9 and TW2.6 cells by using their own stable conditioned medium (CM+ from TIMP3 stable clones and CM− from control clones) as a chemoattractant. CM+ in the bottom chamber reduced the migration (63% in SCC9 and 60% in TW2.6) and invasion (55% in SCC9 and 67% in TW2.6) abilities (Fig. 3e). Furthermore, the TIMP3 recombinant protein reduced the migration (50% in SCC9 and 40% in TW2.6) and invasion (39% in SCC9 and 47% in TW2.6) abilities (Fig. 3f). Moreover, the knockdown of TIMP3 recovered the migration and invasion abilities of SCC9-T9 and TW2.6-T18 clones (Fig. 3g). We also analyzed the migration and invasion abilities after demethylation by 5-aza; data revealed that motility, migration, and invasion all decreased after the treatment (Fig. S2).

### Overexpression of TIMP3 increased cell size and adhesion ability as well as regulated EMT

**Fig. 4:**
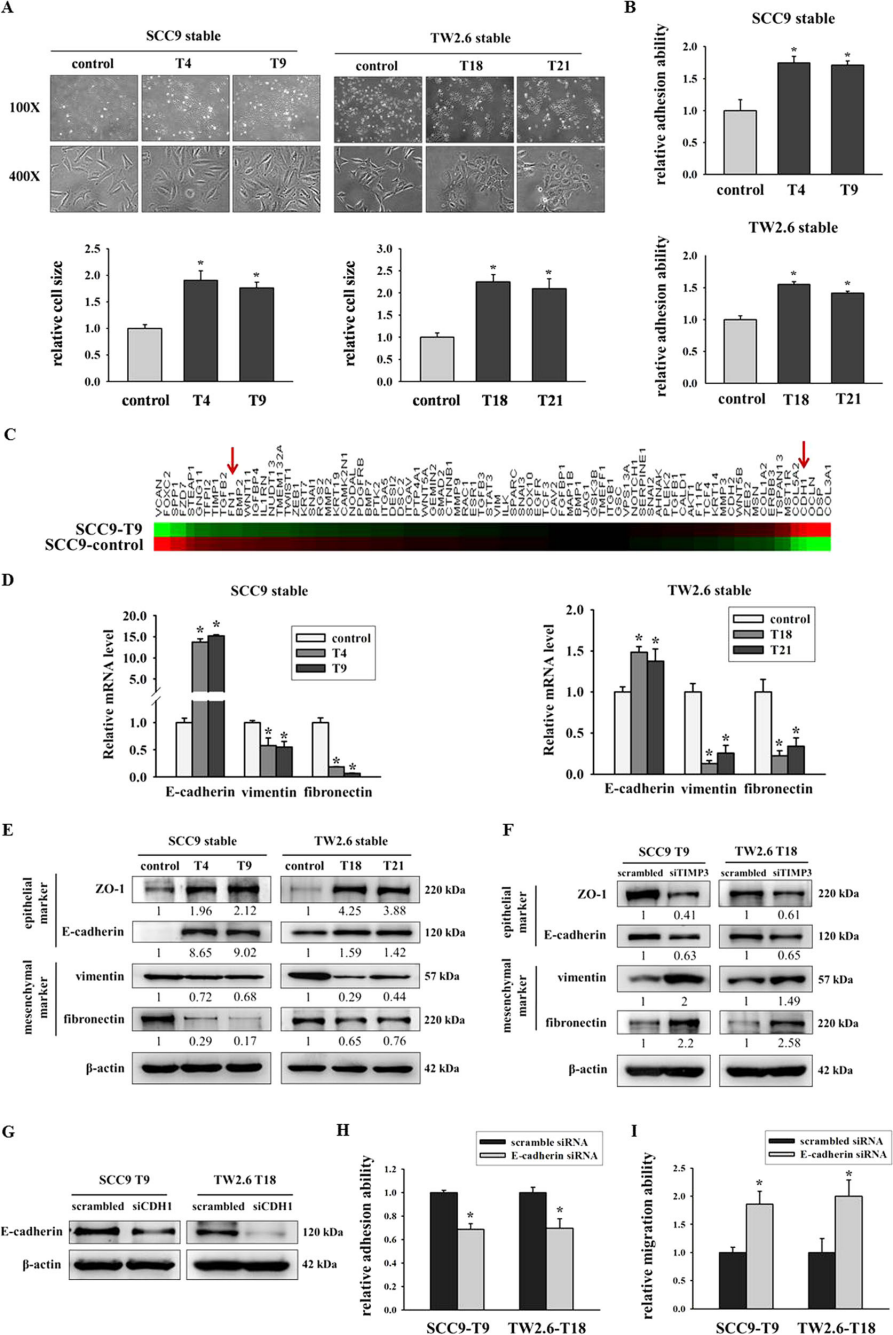
Overexpression of TIMP3 increased cell size and adhesion ability as well as regulated EMT. **a** Morphology and cell size of SCC9 and TW2.6 stable clones. *p < 0.05 compared with control cells. **b** Adhesion assays of oral stable cells were performed by seeding cells for 30 min on plates coated with collagen. *p < 0.05 com*p*ared with control cells. **c** Heat map including 84 EMT-related genes in SCC9-control and SCC9-T9 cells was assessed by Human OneArray^®^. Red arrows indicate the downregulation of fibronectin (FN1) and upregulation of E-cadherin (CDH1) in TIMP3 overexpression SCC9-T9 cells. **d** EMT markers of stable clones were analyzed by real-time PCR. The relative mRNA expression was normalized to GAPDH. *p < 0.05 compared with the control cells. **e** Western blot of EMT-related protein expression. β-actin was used as loading control. **f** Western blot of EMT-related protein expression after transfection of scrambled siRNA or TIMP3 siRNA. β-actin was used as loading control. **g** Knockdown of E-cadherin by siRNA. **h** Adhesion ability of TIMP3 stable cells after E-cadherin knockdown for 2 days. **i** Migration ability of TIMP3 stable cells after E-cadherin knockdown for 2 days

EMT is the mechanism through which epithelial cancers progress toward more aggressive phenotypes with increased cell motility and invasion ability^24,25^. Therefore, we hypothesized that the upregulation of TIMP3 may promote EMT in oral cancer cells. The restoration of TIMP3 substantially changed the visible cell morphology, causing increased clustering of cells into epithelial-like islands with higher degrees of adherence between neighboring cells and decreased fibroblast-like morphology. Moreover, TIMP3 overexpression resulted in an increase in the cell area compared with the control (Fig. 4a), and an increased cell size correlated with the transition to an epithelial cell morphology and a less motile phenotype^26^. In a previous study, TIMP3 was reported to modulate the adhesion ability in thyroid tumor cells^27^. Subsequently, we examined the adhesion ability after TIMP3 overexpression in SCC9 and TW2.6 stable clones. Results demonstrated that TIMP3 increased the cell adhesion ability in TIMP3 stable clones (Fig. 4b), and the knockdown of TIMP3 decreased the adhesion ability in SCC9-T9 and TW2.6-T18 clones (Fig. S3). To elucidate possible molecular pathways underlying the connection between TIMP3 expression and the EMT process, a cDNA microarray was used for analysis. The top 10 differentially expressed EMT-related genes (up- and down-regulation, respectively) are shown in Supplementary Table S3. We found that TIMP3 overexpression in SCC9 stable clones increased E-cadherin (CDH1) expression and reduced fibronectin (FN1) expression (Fig. 4c). Real-time PCR and Western blot assay were also used to confirm the array data. As shown in Fig. 4d, TIMP3 overexpression increased E-cadherin expression, but decreased vimentin and fibronectin expression by real-time PCR assay (Fig. 4d). Moreover, TIMP3 overexpression increased E-cadherin and ZO-1 expression, but decreased vimentin and fibronectin expression by using western blot assay (Fig. 4e). TIMP3 knockdown reduced the expression of epithelial markers (ZO-1 and E-cadherin) and reversed the expression levels of the mesenchymal markers (vimentin and fibronectin) (Fig. 4f). We investigated the contribution of E-cadherin to oral cancer by using E-cadherin siRNA (Fig. 4g). Results suggested that the knockdown of E-cadherin in TIMP3–overexpressed cells decreased the cell adhesion ability (Fig. 4h) and increased the migration ability (Fig. 4i).

### Transcription factors Snail and Twist are required for TIMP3-mediated EMT

**Fig. 5:**
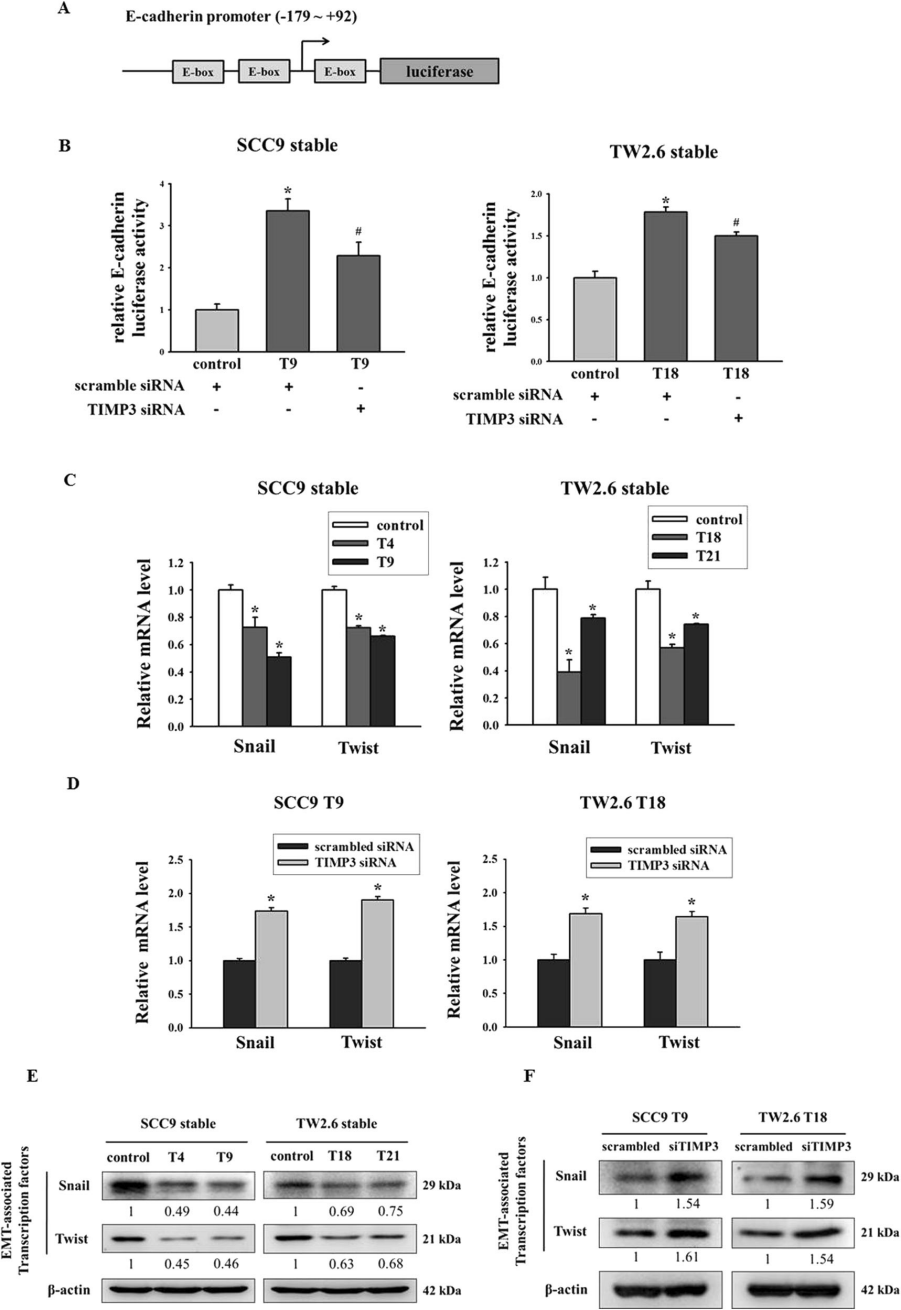
Transcription factors Snail and Twist are required for TIMP3-mediated EMT. **a** The E-cadherin promoter contains three E-box sites was cloned into the luciferase reporter vector. **b** The E-cadherin promoter activity of SCC9 and TW stable cells after transfection of scrambled siRNA or TIMP3 siRNA. *p < 0.05 compared to control stable cell treated with scrambled siRNA. ^#^*p* < 0.05 com*p*ared to TIMP3 stable cell treated with scrambled siRNA. **c** The mRNA levels of EMT-related transcription factors in TIMP3 stable clones. The relative mRNA expression was normalized to GAPDH. *p < 0.05 compared with the control cells. **d** The mRNA levels of EMT-related transcription factors of TIMP3 stable cells after transfection with scrambled siRNA or TIMP3 siRNA. The relative mRNA expression was normalized to GAPDH. *p < 0.05 compared with the scrambled siRNA. **e** The protein expression of EMT-related transcription factors. β-actin was used as loading control. **f** The protein expression of EMT-related transcription factors after transfection with scrambled siRNA or TIMP3 siRNA. β-actin was used as loading control

To determine the contribution of TIMP3 to EMT, a 271-bp (−179 to +92) E-cadherin promoter fragment that contained three E-box sites was cloned into a luciferase vector (Fig. 5a). The luciferase assay results indicated that E-cadherin promoter activity was upregulated in a TIMP3 expression clone and inhibited by TIMP3 knockdown (Fig. 5b). Transcription factors, namely Snail, Slug, and Twist, can bind to E-box sites to regulate target genes^28^. To gain further insights into how TIMP3 regulates EMT, we identified transcription factors whose expression is regulated by TIMP3. Real-time PCR and western blot were used to analyze these transcription factors. Among the transcription factors examined, the mRNA and protein levels of Snail and Twist were decreased in clones overexpressing TIMP3 (Fig. 5c, e). Furthermore, TIMP3 knockdown recovered the expression of Snail and Twist in SCC9-T9 and TW2.6-T18 cells (Fig. 5d, f). These results demonstrate that TIMP3 mediates EMT expression by targeting the transcription factors Snail and Twist.

### Ras-ERK pathway is required for TIMP3 to regulate EMT, migration, and invasion in oral cancer

**Fig. 6:**
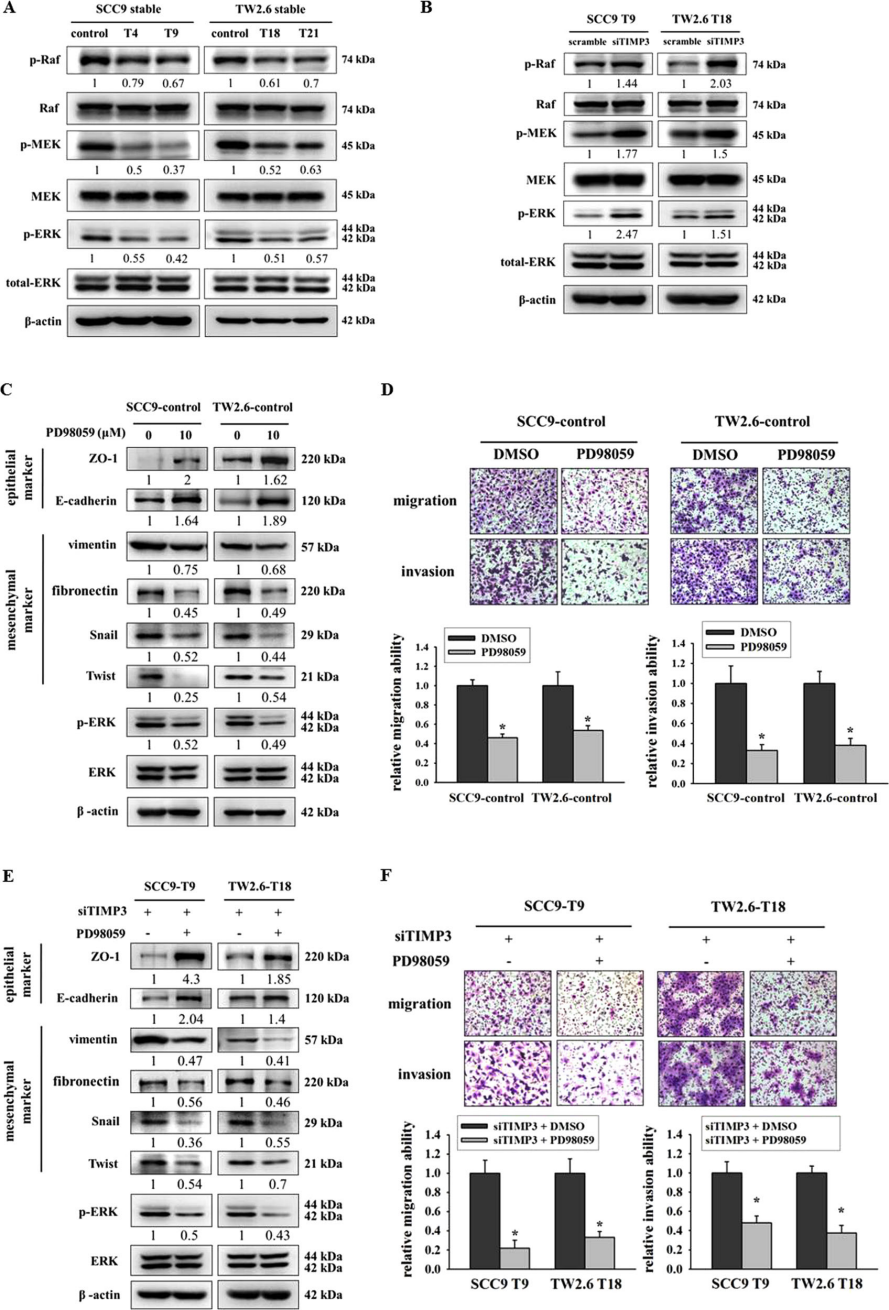
Ras-ERK pathway is required for TIMP3 to regulate EMT, migration, and invasion in oral cancer. **a** The protein expression of EMT-related signaling pathway in oral stable cells. β-actin was used as loading control. **b** The protein expression of EMT-related signaling pathway in TIMP3 stable cells after transfection of scrambled siRNA or TIMP3 siRNA. β-actin was used as loading control. **c** EMT-related protein expression after treatment of PD98059 for 48 h. β-actin was used as loading control. **d** Migration and invasion abilities after treatment of PD98059 for 48 h. *p < 0.05 compared with DMSO. **e** EMT-related protein expression after transfection of TIMP3 siRNA for 24 h and treatment of PD98059 for another 24 h. β-actin was used as loading control. **f** Migration and invasion abilities after transfection of TIMP3 siRNA for 24 h and treatment of PD98059 for another 24 h. *p < 0.05 compared to treatment with TIMP3 siRNA and DMSO

The activation of Ras-ERK signaling triggers the expression of EMT-promoting factors;^29^ therefore, we hypothesized that the regulation of EMT by TIMP3 is dependent on ERK. First, we analyzed the Ras-ERK pathway in our stable clones. Results showed that the restoration of TIMP3 suppressed the activation of p-Raf, p-MEK, and p-ERK in TIMP3 stable clones (Fig. 6a), and the knockdown of TIMP3 in TIMP3 stable clones activated the expression of p-Raf, p-MEK, and p-ERK (Fig. 6b). Next, we used an ERK inhibitor, PD98059, to suppress the ERK pathway in a stable control. Results revealed that the blocking of the ERK pathway by PD98059 upregulated epithelial markers and downregulated mesenchymal markers (Fig. 6c). Moreover, migration and invasion abilities were also decreased after blocking the ERK pathway (Fig. 6d). Then, SCC9-T9 and TW2.6-T18 were transfected with TIMP3 siRNA before treating with PD98059. Results revealed that PD98059 reversed TIMP3 suppression-mediated EMT by upregulating epithelial markers and downregulating mesenchymal markers (Fig. 6e) and further inhibited cell migration and invasion abilities (Fig. 6f). Taken together, these results demonstrated that the regulation of EMT, migration, and invasion by TIMP3 is at least partially dependent on ERK.

### TIMP3 suppressed lymph node metastasis in a TW2.6 orthotopic graft model

**Fig. 7:**
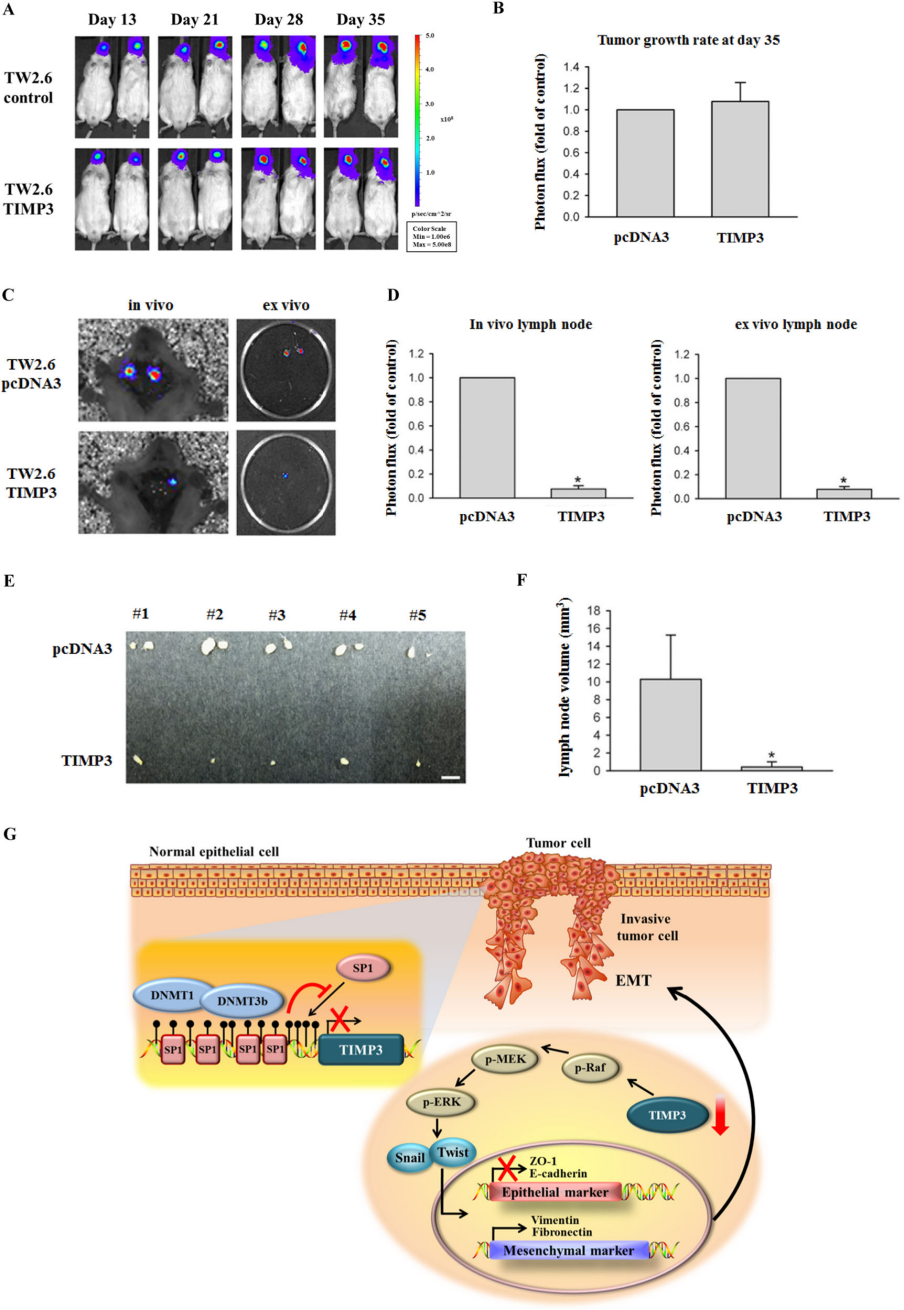
TIMP3 suppressed lymph node metastasis in a TW2.6 orthotopic graft model. **a** Luciferase activity image of mice after injecting with luciferase-tagged TW2.6/pcDNA3 or TW2.6/TIMP3 cells. **b** After 35 days of tumor cell injection, tumors from six mice injected with TW2.6/pcDNA3 or TW2.6/TIMP3 were quantified by measuring the photon influx. **c**, **d** Lymph node metastasis was imaged at the end of the study with the mean signal for each group indicated (*n* = 6). *p < 0.05 compared with the TW2.6/pcDNA3 groups. **e**, **f** Macroscopic analysis of neck lymph nodes. The appearance, number, and volume of neck lymph nodes were photographed, enumerated, and measured after removal. *p < 0.05 compared with the TW2.6/pcDNA3 groups. **g** Proposed model for the role of TIMP3 methylation contributes to oral cancer metastasis

The aforementioned results indicated that TIMP3 inhibited migration and invasion and induced EMT in oral cancer cells. To further evaluate whether TIMP3 overexpression affects metastasis in vivo, luciferase-expressing TW2.6-Luc cells were established to analyze tumor growth and metastasis. No significant differences existed between control TW2.6 cells (TW2.6/pcDNA3-Luc) and TW2.6/TIMP3-Luc in tumor growth after cells were injected into mice at 35 days (Fig. 7a, b). Mice were sacrificed at the end of the experiment; in vivo or ex vivo images of their neck lymph nodes revealed a lower intensity in TW2.6/TIMP3-Luc-injected mice than in TW2.6/pcDNA3-Luc-injected mice (Fig. 7c, d). Most mice developed neck lymph node metastasis within 35 days after cancer cell injection; we further determined the frequency of neck lymph node metastasis and the volume of lymph nodes excised from the TW2.6/pcDNA3 and TW2.6/TIMP3 groups. The volume of neck metastatic lymph nodes significantly decreased in TW2.6/TIMP3 mice than in TW2.6/pcDNA3 mice (Fig. 7e, f).

## Discussion

Loss of TIMP3 has been reported in numerous human cancers, including thyroid, prostate, and colorectal cancers^13,27,30^. In this study, we observed a significant downregulation of TIMP3 expression in oral cancer tissues compared with adjacent normal tissues, suggesting that the loss of TIMP3 might be a critical event in the pathogenesis of oral cancer. Moreover, in vitro data demonstrated that TIMP3 mRNA and protein levels were downregulated in oral cancer cell lines compared with normal oral cell lines (HOK and SG). Similar results were reported in other types of cancer cell lines, such as hepatocellular carcinoma cell lines^31^.

Loss or downregulation of TIMP3 expression has been linked to TIMP3 gene methylation in esophageal adenocarcinoma, gastric cancer, and non–small-cell lung cancer^32–34^. Although our results did not reveal the definite sequence of DNA methylation that is responsible for TIMP3 suppression, loss of TIMP3 protein expression in human gastric cancer cell lines is closely correlated with the hypermethylation of TIMP3 in the region near the transcription start site (−116 to 64)^33^. Nevertheless, transcriptional repression by DNA methylation involves the inhibition of binding of transcription factors to the active region of the gene promoter, and Sp1 is one of the major transcription factors that enhanced the promoter activity of TIMP3, which has been reported in chondrocytes and glioblastoma^35,36^. Consistently, we found that DNA methylation inhibited the binding of the transcription factor Sp1 to a region of the TIMP3 promoter that was active when DNA methylation was absent. Moreover, inhibition of the association between Sp1 and the fragment blocked upregulation of TIMP3 in response to 5-aza. In conclusion, these findings indicate that Sp1 is a crucial transcription factor that activates TIMP3 transcription when TIMP3 DNA methylation is absent.

DNMTs are key enzymes involved in DNA methylation and are frequently upregulated during tumorigenesis^37^. The DNMT family can be divided into two major groups: maintenance methyltransferases, such as DNMT1, and de novo methyltransferases, such as DNMT3A and DNMT3B. In colorectal carcinoma, knockout of both DNMT1 and DNMT3B caused promoter demethylation of TIMP3, thus increasing the TIMP3 mRNA level^38^. Therefore, we mainly investigated the effects of DNMT1 and DNMT3B on the expression of TIMP3 in this study. After scanning our oral cell lines, we observed that the mRNA expression of DNMT1 and DNMT3B was higher in most cancer cell lines compared with normal cell lines. Moreover, data from the TCGA database suggest that mRNA expression of DNMT1 and DNMT3B is upregulated in HNSCC tissues. Tumor suppressor genes may be epigenetically silenced by both DNMT1 and DNMT3B^39,40^. In the present data, knockdown of DNMT1 or DNMT3B restored the expression of TIMP3 in the oral cancer cell lines. Using DNMT inhibitors, we confirmed that when DNMTs are removed from the promoter, the ability of Sp1 to bind to the TIMP3 promoter improved.

To determine the contribution of TIMP3 to oral carcinogenesis, we restored TIMP3 expression in SCC9 and TW2.6 oral cancer cell lines. Different results for the effect of TIMP3 expression on tumor cell growth have been reported. No effect was observed on the in vitro proliferation of human leukemia and thyroid tumor cells;^27,41^ by contrast, TIMP3 induced cell apoptosis in human melanoma and prostate cancer^13,42,43^. In our study, TIMP3 expression did not affect cell growth compared with control cells. Nevertheless, we demonstrated that TIMP3 plays a crucial role in regulating migration and invasion processes in oral cancer. TIMP3 restoration in oral cancer cell lines reduced wound healing, migration, and invasion abilities. Moreover, the recombinant protein of TIMP3 or the condition medium from TIMP3 stable cells reduced cancer cell migration and invasion abilities, and knockdown of TIMP3 expression in TIMP3 stable cells recovered cell migration and invasion abilities, which were used to confirm the aforementioned results. In an animal model, TIMP3 restoration inhibited leukemia cell growth and angiogenesis in nude mice^41^, and the metastatic dissemination of melanoma and lymphoma cells to multiple organs was enhanced in TIMP3^−/−^ mice^44^. To gain further insights into how TIMP3 regulates oral cancer metastasis in vivo, an orthotopic graft model was used. The results of the present study showed that TIMP3 restoration in oral cancer cells dramatically reduced metastasis to lymph nodes with no effect on primary tumor growth.

EMT is a key step during tumor invasion and metastasis, and several molecular pathways that mediate EMT in cancer cells have been identified^24,45,46^. A study indicated that TIMP3 increased the cell adhesion ability in thyroid tumors^27^. In addition, TIMP3 overexpression rescued EMT-related marker expression induced by TGF-β in gastric cancer cells^47^. We observed that TIMP3 changed the cell morphology from fibroblastic- to epithelial-like islands and increased the cell adhesion ability in oral cancer. Our microarray analyses suggested that TIMP3 can function as an inhibitor of EMT, directly regulating the expression of several genes involved in EMT. TIMP3 restoration increased the cell adhesion ability by upregulating the epithelial markers (ZO-1 and E-cadherin) and downregulating the mesenchymal markers (vimentin and fibronectin). Moreover, knockdown of TIMP3 in TIMP3 stable cells activated the EMT process by reducing epithelial markers and increasing mesenchymal markers.

The transcription factors Snail, Slug, Twist, and ZEB1/2 contribute to the regulatory network during EMT^29,48^. In this study, TIMP3 suppression enhanced the expression of Snail and Twist in oral cancer cells. Furthermore, Snail and Twist expression was negatively correlated with that of E-cadherin. Because EMT is one of the pathways mediated by mitogen-activated protein kinase signaling or the PI3K/AKT signaling pathway^49,50^, EMT may be regulated by TIMP3 through these signaling pathways. In the present study, TIMP3 inhibited the expression of p-ERK, and the ERK pathway was restored after the knockdown of TIMP3. These findings suggest that TIMP3 may suppress EMT by regulating Snail and Twist through the ERK signaling pathway.

In summary, we identified TIMP3 as a clinical marker for predicting oral cancer. In addition, TIMP3 expression was regulated by promoter hypermethylation, and loss of TIMP3 may be due to the blocking of Sp1 binding to the TIMP3 promoter as well as the upregulation of DNMT1 and DNMT3B. Moreover, TIMP3 regulated EMT by increasing the expression levels of the epithelial markers (ZO-1 and E-cadherin) and reducing the expression levels of the mesenchymal markers (vimentin, fibronectin, Snail, and Twist) through the Ras-ERK pathway (Fig. 7g). In conclusion, these results suggest that the suppression of TIMP3 by DNA methylation contributes to oral cancer metastasis.

## Materials and methods

### Patient specimens

In all, 25 pairs of oral cancer tissues and their corresponding normal tissues were obtained from Chung Shan Medical University Hospital in Taichung, Taiwan. Patient specimens were immediately frozen in liquid nitrogen after surgery. In this study, 17 pairs of tissues were used to analyze mRNA expression and another 8 pairs of tissues were used for Western blot. For real-time PCR and western blot analysis, human tissues will be placed in a mortar containing liquid nitrogen and then ground in a mortar to produce a fine powder. Then the powder was dissolved to the appropriate buffer according to different experiments as previously described^51^.

### Cell and cell culture

Human oral squamous cell carcinoma cell lines, including SCC9 cells were purchased from the American Type Culture Collection (ATCC, Manassas, VA) and were authenticated by STR profiling. SCC9 cell lines were cultured in appropriate medium supplemented with 10% FBS. Human oral gingival cells (SG) were cultured in DMEM medium supplemented with 10% FBS. Primary human oral keratinocytes (HOK) were cultured in Gibco Keratinocyte-SFM. All cell cultures were maintained at 37 °C in a humidified atmosphere of 5% CO_2_.

### Western blot analysis

The cell lysates was separated in a 10% polyacrylamide gel and transferred onto a nitrocellulose membrane. The blot was subsequently incubated with 5% nonfat milk in Tris-buffered saline (20 mM Tris, 137 mM NaCl, pH 7.6) for 1 h to block non- specific binding and then overnight with antibodies against TIMP3, Sp1, E-cadherin, ZO-1, vimentin, fibronectin, Snail, Twist, p-Raf, Raf, p-MEK, MEK, p-ERK, ERK, and β-actin. TIMP3 (MAB3318) antibodies were purchased from Millipore (Billerica, MA, USA). E-cadherin (610182) antibodies were purchase from BD Biosciences (San Jose, CA, USA). SP1 (sc-59), ZO-1 (sc-10804), vimentin (sc-6260) and fibronectin (sc-9068) antibodies were purchased from Santa Cruz Biotechnology (Santa Cruz, CA, USA). p-Raf (#9427), Raf (#9422), p-MEK (#9121), MEK (#9122), p-ERK (#4370), ERK (#9102) and Snail (#3879) antibodies were purchased from Cell Signaling Technology (Danvers, MA, USA). β-actin (ab8226) antibodies were purchase from Abcam (Cambridge, UK). Afterwards, signal was detected by using enhanced chemiluminescence (ECL) commercial kit (Amersham Biosciences) and relative photographic density was quantitated by scanning the photographic negatives on a gel documentation and analysis system (AlphaImager 2000, Alpha Innotech Corporation,San Leandro, CA, USA).

### RT-PCR, real-time PCR, and microarray

Total RNA was extracted from oral tissues and oral cell lines using Total RNA Mini Kit (Geneaid). Total RNA was reverse transcribed into cDNA by SuperScript III First-Strand Synthesis Supermix (Invitrogen, Carlsbad, CA). The PCR was performed in a reaction mixture containing 2 μL cDNA, 0.2 mM dNTP mixture, 2 mM of each primers, 1 U Taq DNA polymerase, and 1-fold concentration of Thermal Pol Buffer (New England BioLabs, MA, USA) by denaturation at 95 °C for 5 min, followed by amplification of indicated cycles of 95 °C for 30 s, 60°C for 30 s, and 72 °C for 60 s. The specific primer sequences for these genes are as following: TIMP3: 5′- CTGACAGGTCGCGTCTATGA-3′ (forward), 5′-GGCGTAGTGTTTGGACTGGT-3′ (reverse), and GAPDH: 5′-CGGAGTCAACGGATTTGGTCGTAT-3′ (forward), 5′- AGCCTTCTCCATGGTGGTGAAGAC-3′ (reverse). In quantitative real-time PCR, TIMP3, DNMT1, DNMT3B, E-cadherin, vimentin, fibronectin, Snail, Twist, and GAPDH expressions were determined using Power SYBR Green PCR Master Mix (Applied Biosystems). Cycling conditions were: 10 min at 95 °C followed by 50 repeats of the following cycle: 95 °C for 15 s, annealing at the appropriate temperature for 60 s. GAPDH expression was used for normalization of target gene expression. The detailed information of primer was showed in Supplementary Table S1. For cDNA microarray, RNA isolated from SCC9-control or SCC9-T9 was submitted to the Phalanx Biotech Group (Hsinchu, Taiwan) for expression profiling and analysis.

### Pyrosequencing

Genomic DNA was isolated from oral cell lines. Sufficient quality and quantity of extracted DNA was bisulfite-modified and subjected to Pyrosequencing. PCR primers and sequencing primer for pyrosequencing were all designed by Genomics according to TIMP3 promoter region. The detailed information of primer was showed in Supplementary Table S2. Results were provided by Genomics and were represented as a percentage of methylation level.

### Construction of TIMP3 expression vector

The TIMP3 cDNA was isolated by RT-PCR from a human blood cDNA using the following primers: 5′-GAATTCCAGCGGCAATGACCCCTTG-3′ (forward) and 5′-GGATCCGCGCTCAGGGGTCTGTGG-3′ (reverse), containing the EcoRI and the BamHI restriction sites, respectively. The 636 nucleotides PCR product was digested with EcoRI and BamHI endonucleases and inserted into the pcDNA3.

### Treatment with DNMT inhibitor and TIMP3 recombinant protein

Twenty-four hours prior to treatment, cells were plated at 5 × 10^5^ cells/6 cm plate. Treatments consisted of 5-aza-2′-deoxycytidine (5-aza; Sigma) for 96 h. Drug levels were maintained by replacing medium containing the relevant concentration of drug every 24 h. After treatment with 5-aza, SCC9 and TW2.6 were used for wound healing assay, migration assay, invasion assay, real-time PCR, and ChIP assay. The recombinant TIMP3 protein (R&D Systems, Minneapolis, MN, USA) was used at 50 nM. After treatment with recombinant TIMP3 protein, SCC9 and TW2.6 were used to analyze the migration and invasion abilities.

### Cell proliferation assay

Cell proliferation was analyzed by an MTT assay as described previously^52^. Briefly, oral cancer cells were seeded in 24-well plates at appropriate density and cell proliferation was examined every 24 h from 1 to 6 day. The results of proliferation are reported as fold increase relative to first day value set as 1.

### Wound healing assay

SCC9 stable clones (9 × 10^5^ cells) and TW2.6 stable clones (1.5 × 10^6^ cells) were plated in 6 cm plates for 24 h, wounded by scratching with a pipette tip, and then incubated with medium containing 0.5% FBS. Cells were photographed using a phase-contrast microscope.

### Migration and invasion assay

In the migration assay, SCC9 stable clones (3 × 10^4^ cells) and TW2.6 stable clones (5 × 10^4^ cells) were seeded in the upper chamber of 24-well Transwell inserts (Millipore, Bedford, MA, USA) in serum-free medium. After 24 h of incubation at 37 °C, filters were fixed with methanol and stained with Giemsa stain (Sigma). Migrated cells were counted under an inverted microscope in six randomly chosen fields.

For invasion assay, 60 μl Matrigel (25 mg/50 ml; BD Biosciences, MA, USA) was added to Transwell inserts. SCC9 stable clones (3 × 10^4^ cells) and TW2.6 stable clones (5 × 10^4^ cells) were seeded in the upper chamber of Matrigel-coated Transwell inserts in serum-free medium. After 48 h of incubation at 37 °C, filters were fixed with methanol and stained with Giemsa stain (Sigma). Invasive cells were counted under an inverted microscope in six randomly chosen fields.

### Adhesion assay

TIMP3 stable cells and control cells were seeded in 24-well plate pretreated with collagen (10 μg/ml, 300 μL/well). After 30 min of incubation, non-attached cells were removed by gently washing twice with 1X PBS. Attached cells were fixed with methanol for 20 min at room temperature, followed by staining in crystal violet for 30 min. Stained cells were lysed by destain buffer and the intensity of stain was quantified by a spectrometer at the absorbance of 590 nm.

### RNA interference experiments

The human small interfering ribonucleic acids (siRNA) for TIMP3, E-cadherin, Sp1, DNMT1, DNMT3B, and scrambled siRNA were obtained from Ambion Inc. Cells were transfected with siRNA using Lipofectamine RNAiMAX reagent (Invitrogen).

### Luciferase assay

A density of 8 × 10^4^ cells per well was plated in 24-well plates for 24 h. The pGL3-control vector, pGL3-basic vector, pGL3-TIMP3 vector (−940 to +376), and pGL3-CDH1 vector (−179 to +92) were co-transfected with a β-galactosidase expression vector (pCH110) into cells using Lipofectamine 2000 (Invitrogen). After 24 h of transfection, cell lysates were harvested, and luciferase activity was determined using a luciferase assay kit. The value of the luciferase activity was normalized to transfection efficiency and monitored by β-galactosidase expression.

### Chromatin immunoprecipitation analysis

Chromatin immunoprecipitation analysis (ChIP) was performed as described previously^53^. DNA immunoprecipitated with antibodies specific to Sp1, DNMT1, DNMT3B, and rabbit immunoglobulin G was purified and extracted. Immunoprecipitated DNA was analyzed by RT-PCR using specific primers according to previous study^36^. The primers used for PCR to amplify the TIMP3 promoter encompassing the Sp1 binding sites were TIMP3 Fw, 5′-CCACGGCGGCATTATTCCCTATAA-3′, TIMP3 Rev, 5′-AGGAGCAAGAGGAGGAGGAGAA-3′.

### Orthotopic implantation

All animal experiments were performed according to the protocol approved by the Institutional Animal Care and Use Committee of Chung Shan Medical University. Six age-matched male severe combined immunodeficient (SCID) mice were used in assays for tumor growth and lymph node metastasis. TIMP3-expressing luciferase-tagged TW2.6 cells (5 × 10^5^ cells) and control TW2.6 cells (5 × 10^5^ cells) were injected into the palate of SCID mice. The tumor formation and lymph node metastasis were monitored by non-invasive bioluminescent imaging system (Xenogen IVIS-200 system) after injection and then sacrificed at day 35 to quantify the volume of metastatic neck lymph nodes.

### Database analysis

HNSCC tissues with clinical information from the Cancer Genome Atlas **(**TCGA) database were used to analyze the expression of DNMT1 and DNMT3B between cancer and normal tissues. The methylation levels of TIMP3 CpG island data were analyzed from a database of DNA methylation and gene expression in human cancer (MethHC). The position of TIMP3 CpG islands was predicted by MethPrimer.

### Statistical analysis

All experiments were done in triplicate, and results were reported as mean ± standard deviation (SD). Statistical significances of difference in this study were calculated by Student’s *t*-test (Sigma-Stat 2.0, Jandel Scientific, San Rafael, CA, USA). *P* value < 0.05 was considered significant.

## Supplementary information

Supplementary Figure 1

Supplementary Figure 2

Supplementary Figure 3

Supplementary data

Supplementary Figure Legends
