# Supplementary figures and images for: Loss of TIMP3 by promoter methylation of Sp1 binding site promotes oral cancer metastasis
Source: Cell Death Dis. 2019 Oct 17;10(11):793. doi: 10.1038/s41419-019-2016-0 (PMC6797751; doi:10.1038/s41419-019-2016-0)

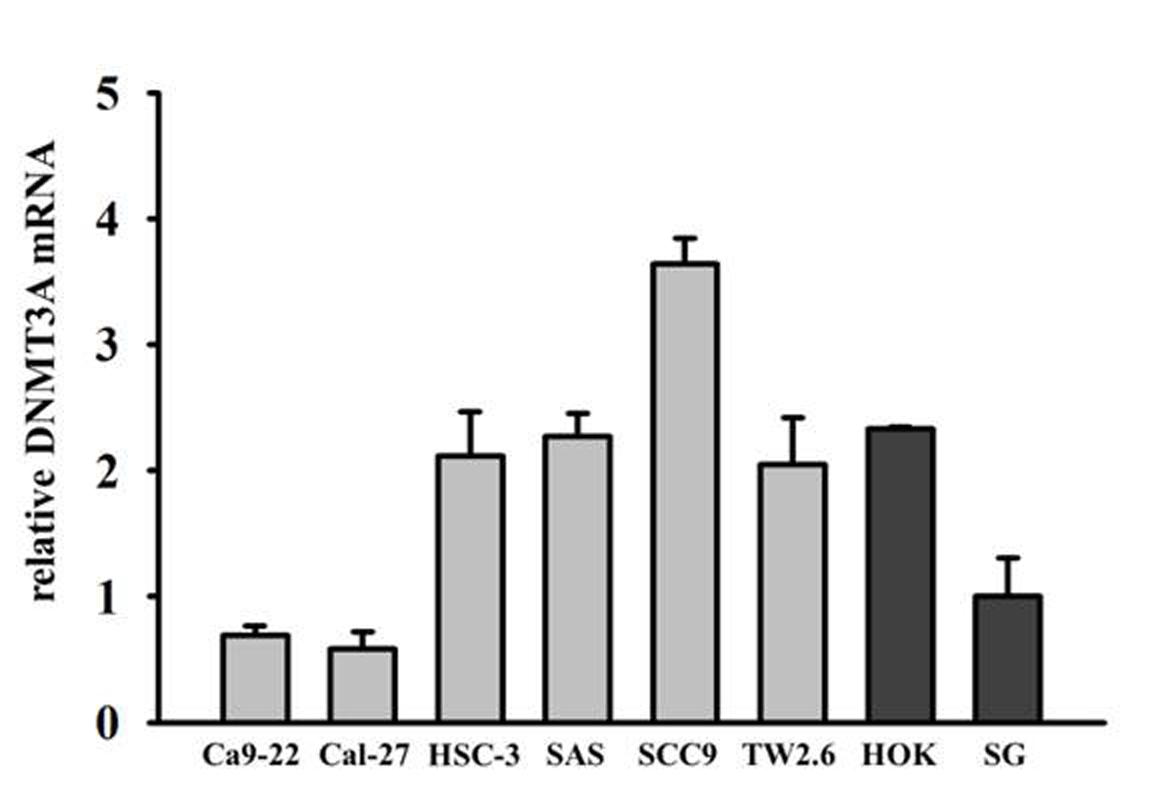

Supplement: Supplementary file 1 — Supplementary Figure 1 [file 41419_2019_2016_MOESM1_ESM.tif]

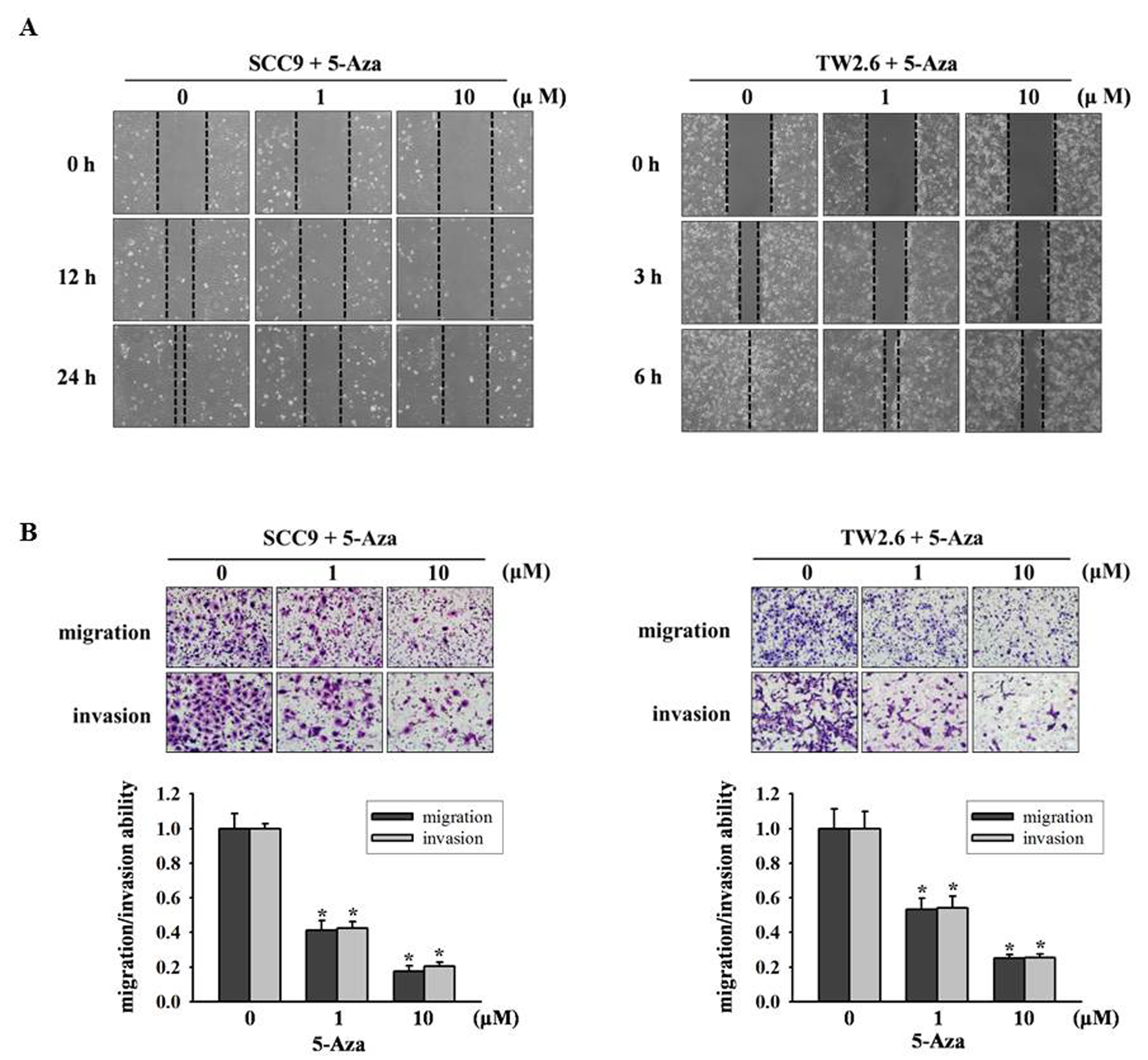

Supplement: Supplementary file 2 — Supplementary Figure 2 [file 41419_2019_2016_MOESM2_ESM.tif]

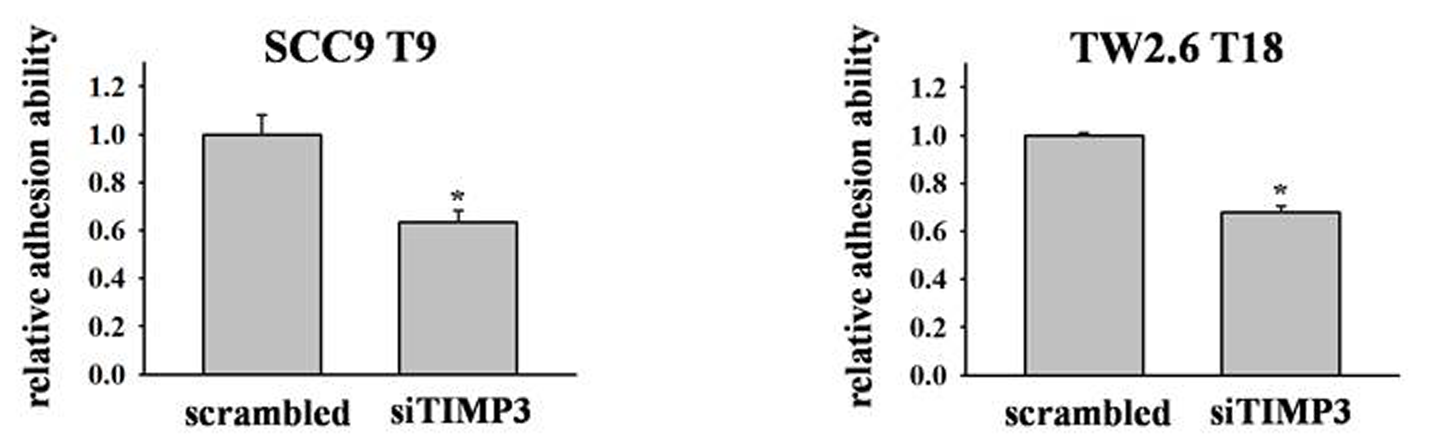

Supplement: Supplementary file 3 — Supplementary Figure 3 [file 41419_2019_2016_MOESM3_ESM.tif]
