# Supplementary material for: Loss of TIMP3 by promoter methylation of Sp1 binding site promotes oral cancer metastasis
Source: Cell Death Dis. 2019 Oct 17;10(11):793. doi: 10.1038/s41419-019-2016-0 (PMC6797751; doi:10.1038/s41419-019-2016-0)
Supplement: Supplementary file 4 — Supplementary data [file 41419_2019_2016_MOESM4_ESM.docx]

**Table S1. Primer sets used for SYBR real-time PCR**

| **Genes** | **Forward primer (5’-3’)** | **Reverse primer (5’-3’)** |
| --- | --- | --- |
| GAPDH | GCTCCTCCTGTTCGACAGTCA | GCGCCCAATACGACCAAAT |
| β2-MG | GCTATCCAGCGTACTCCAAA | GATGGATGAAACCCAGACAC |
| TIMP3 | CTGACAGGTCGCGTCTATGA | GGCGTAGTGTTTGGACTGGT |
| DNMT1  DNMT3B  E-cadherin  vimentin  Fibronectin  Snail  Twist | GCTGTGCCCGTCTGGCTGAG  GGCAAGTTCTCCGAGGTCTCTG  TGAAGGTGACAGAGCCTCTGGAT  GACAATGCGTCTCTGGCACGTCTT  GGGAGCCTCGAAGAG  GAGGCGGTGGCAGACTAG  CGGGAGTCCGCAGTCTTA | TTCCGTGGGCGTTTCACGGG  TGGTACATGGCTTTTCGATAGGA  TGGGTGAATTCGGGCTTGTT  TCCTCCGCCTCCTGCAGGTTCTT  AACAAGTACAAACCAACGCA  GACACATCGGTCAGACCAG  TGAATCTTGCTCAGCTTGTC |

GAPDH: glyceraldehyde-3-phosphate dehydrogenase. β2-MG: β2-microglobulin DNMT: DNA methyltransferase.

**Table S2. Primer used for pyrosequencing**

| **Primer names** | **Sequences (5’-3’)** |
| --- | --- |
| P1 PCR forward primer | TGGGTGGGTGTTAGTTGG |
| P1 PCR reverse primer | CCCCCCCCCTCAAACCAATAAC |
| P1-a sequencing primer | GGGAGAGTTTTAGTTTATTTAT |
| P1-b sequencing primer  P2 PCR forward primer  P2 PCR reverse primer  P2-c sequencing primer | GGTTTAGTTTAGTTATTTTTTGTT  TTTTGGAGGGTAGATGAGGTAATG  AAACTACTACTCCCCTCTCCAAAATT  GTAATGAGGTTTTGTTATTGGTT |

**Table S3. Top 10 up- and down-regulation of EMT-related genes in TIMP3 overexpression SCC9-T9 cell**

| **Gene symbol** | **Protein name** | **Log2 (fold change)** | **change** |
| --- | --- | --- | --- |
| COL3A1 | collagen type III alpha 1 chain | 5.969977944 | Up |
| DSP | desmoplakin | 3.761722115 | Up |
| OCLN | occludin | 2.764833189 | Up |
| CDH1  COL5A2  MST1R  TSPAN13  ERBB3  COL1A2  MSN | cadherin 1  collagen type V alpha 2 chain  macrophage stimulating 1 receptor  tetraspanin 13  erb-b2 receptor tyrosine kinase 3  collagen type I alpha 2 chain  moesin | 2.406750198  2.269277343  1.252004424  1.093769253  0.749695369  0.684459634  0.649720142 | Up  Up  Up  Up  Up  Up  Up |
| VCAN | versican | -3.470625562 | Down |
| FOXC2  SPP1  FZD7  STEAP1  GNG11  TFPI2  TIMP1  TGFB2  FN1 | Forkhead box protein C2  secreted phosphoprotein 1  frizzled family receptor 7  STEAP family member 1  G protein subunit gamma 11  tissue factor pathway inhibitor 2  TIMP metallopeptidase inhibitor 1  transforming growth factor beta 2  fibronectin 1 | -2.848875524  -2.186846857  -2.157076237  -1.532781715  -1.267692195  -1.236307434  -1.139502344  -1.086861596  -1.053601262 | Down  Down  Down  Down  Down  Down  Down  Down  Down |
