# Supplementary material for: Loss of TIMP3 by promoter methylation of Sp1 binding site promotes oral cancer metastasis
Source: Cell Death Dis. 2019 Oct 17;10(11):793. doi: 10.1038/s41419-019-2016-0 (PMC6797751; doi:10.1038/s41419-019-2016-0)
Supplement: Supplementary file 5 — Supplementary Figure Legends [file 41419_2019_2016_MOESM5_ESM.docx]

**Table S1. Primer sets used for SYBR real-time PCR**

**Table S2. Primer used for pyrosequencing**

**Table S3. Top 10 up- and down-regulation of EMT-related genes in TIMP3 overexpression SCC9-T9 cell**

**Supplementary Figure Legends**

**Figure S1. The mRNA levels of DNMT3A in oral cancer cell lines and normal oral cell lines.**

**Figure S2. DNMT inhibitor 5-aza suppresses motility, migration and invasion ability in oral cancer cells.** (A) Wound healing assay for SCC9 and TW2.6 after treatment with 5-Aza. (B) Migration and invasion assay for SCC9 and TW2.6 after treatment with 5-Aza. *p<0.05 compared with 5-Aza 0 μM group.

**Figure S3.** **TIMP-3 affects adhesion ability in TIMP3 stable cells.** Adhesion assay of SCC9-T9 and TW2.6-T18 after transfection of scrambled siRNA or TIMP-3 siRNA *p<0.05 compared with scrambled siRNA.
